# Supplementary material for: Asynchronous magnetic resonance elastography: Shear wave speed reconstruction using noise correlation of incoherent waves
Source: Magn Reson Med. 2022 Oct 27;89(3):990–1001. doi: 10.1002/mrm.29502 (PMC9792433; doi:10.1002/mrm.29502)
Supplement: Supplementary file 1 — DATA S1 MATLAB code used to generate the numerical simulations of Figure 1 [file MRM-89-990-s002.zip › k-Wave/helpfiles/example_na_controlling_the_pml.html]

Controlling The Absorbing Boundary Layer Example (k-Wave)


# Controlling The Absorbing Boundary Layer Example

The first-order simulation codes included within the k-Wave Toolbox (`kspaceFirstOrder1D`, `kspaceFirstOrder2D`, and `kspaceFirstOrder3D`) use a special type of anisotropic absorbing boundary layer known as a *perfectly matched layer* (PML) to absorb acoustic waves when they reach the edge of the computational domain. By default, this layer occupies a strip around the edge of the domain of 20 grid points in 1D and 2D, and 10 grid points in 3D. Without this boundary layer, the computation of the spatial derivates via the FFT causes waves leaving one side of the domain to reappear at the opposite side. The use of the PML thus facilitates infinite domain simulations without the need to increase in the size of the computational grid. This example demonstrates how to control the parameters of the PML within k-Wave via optional input parameters.

- Open the file in the MATLAB Editor
- Run the file in MATLAB

## Contents

- Controlling the properties of the absorbing boundary layer
- Switching off the PML
- A partially effective PML
- Setting the PML to be outside the computational grid

## Controlling the properties of the absorbing boundary layer

The PML has four properties, each of which can be controlled via optional input parameters:

1. **Size:** The size of the layer on each edge of the domain is set by `'PMLSize'` in units of grid points. The default setting is 20 grid points in 1D and 2D, and 10 grid points in 3D. If the size is specified as a single number, this is used for all Cartesian directions. Alternatively, the size for each direction can be set individually by setting `'PMLSize'` to `[PML_X_SIZE, PML_Y_SIZE]` in 2D, and `[PML_X_SIZE, PML_Y_SIZE, PML_Z_SIZE]` in 3D.
2. **Absorption:** The absorption within the layer is set by `'PMLAlpha'` in units of Nepers per grid point. The default setting is 2 for all dimensions. Again, if the absorption is specified as a single number, this is used for all Cartesian directions. Alternatively, the absorption for each direction can be set individually by setting `'PMLAlpha'` to `[PML_X_ALPHA, PML_Y_ALPHA]` in 2D, and `[PML_X_ALPHA, PML_Y_ALPHA, PML_Z_ALPHA]` in 3D.
3. **Location:** The location of the layer can be set to be *inside* or *outside* the computational grid created by the user by changing the value of the Boolean flag `'PMLInside'` (the default setting is `true`). If `'PMLInside'` is set to `false`, the input grids are enlarged on each edge by the size given by `'PMLSize'`.
4. **Visibility:** The visibility of the absorbing layer within the animations displayed during the simulation is controlled by the Boolean input parameter `'PlotPML'` (the default setting is `true`).

For accurate simulations, care must be taken that the initial pressure distribution and the sensor mask don't lie within the PML. This can be avoided by setting `'PMLInside'` to `false`. However, the computational time will still be dependent on the *total* size of the grid including the PML (i.e., computations will be fastest for grids where the total number of grid points in each dimension is given by a power of two).

## Switching off the PML

The effect of the PML can be illustrated most clearly by switching it completely off by setting the optional input `'PMLAlpha'` to `0` (set `example_number = 1` within the example m-file). This sets the absorption within the layer to zero, thus the waves leaving one side of the domain reappear at the opposite side. A visualisation of the recorded time-series is given below.

A similar effect is seen if the value for `'PMLAlpha'` is set too high (set `example_number = 2` within the example m-file). This causes the waves to be reflected from the layer.

## A partially effective PML

The effectiveness of the PML depends on its size and absorption, but also on the time step used in the simulation (the more time steps the wave spends in the layer, the more it will be absorbed). A partially effective PML can be illustrated by reducing the size of the PML by setting the optional input `'PMLSize'` to `2` (set `example_number = 3` within the example m-file). The layer absorbs some (but not all) of the waves approaching the boundaries, and some of the wave wrapping seen in the previous example is still visible.

## Setting the PML to be outside the computational grid

By default, the PML will be *inside* the grid defined by the user, so care must be taken that the source and sensor are not defined within this region. This can be avoided by setting the optional input `'PMLInside'` to `false` (set `example_number = 4` within the example m-file). In this case, the computational grid and input variables are automatically expanded by the size of the PML. This setting gives several additional command line status readouts, including feedback on expanding the input grids and the total size of the computational grid (in this case 168 by 168 grid points).

```
Running k-Wave simulation...
  start time: 04-Jun-2017 13:10:18
  reference sound speed: 1500m/s
  dt: 20ns, t_end: 12.06us, time steps: 604
  input grid size: 128 by 128 grid points (12.8 by 12.8mm)
  maximum supported frequency: 7.5MHz
  smoothing p0 distribution...
  expanding computational grid...
  computational grid size: 168 by 168 grid points
  calculating Delaunay triangulation...
  precomputation completed in 0.29097s
  starting time loop...
  estimated simulation time 2.718s...
  simulation completed in 3.0234s
  total computation time 3.355s
```

As the inner part of the computation grid is identical to that in the Homogeneous Propagation Medium Example, the recorded time-series (shown below) is also identical. In this case, the default size and absorption for the PML are adequate to prevent any visible wave wrapping. For any particular example, if the performance of the PML is not sufficient, increasing the size of the PML, optimising the value of `PMLAlpha`, or reducing the time-step will give improvements.
